# Roles of PI3K/AKT/mTOR Axis in Arteriovenous Fistula

**DOI:** 10.3390/biom12030350

**Published:** 2022-02-23

**Authors:** Stefano Ratti, Raffaella Mauro, Cristina Rocchi, Sara Mongiorgi, Giulia Ramazzotti, Mauro Gargiulo, Lucia Manzoli, Lucio Cocco, Roberta Fiume

**Affiliations:** 1 Cellular Signalling Laboratory, Department of Biomedical and Neuromotor Sciences, University of Bologna, 40126 Bologna, Italy; stefano.ratti@unibo.it (S.R.); s.mongiorgi@unibo.it (S.M.); giulia.ramazzotti@unibo.it (G.R.); lucia.manzoli@unibo.it (L.M.); roberta.fiume@unibo.it (R.F.); 2 Vascular Surgery Unit, IRCCS University Hospital, Policlinico S. Orsola-Malpighi, 40126 Bologna, Italy; raffaella.mauro@aosp.bo.it (R.M.); cristina.rocchi@studio.unibo.it (C.R.); mauro.gargiulo2@unibo.it (M.G.)

**Keywords:** AVF fistula, PI3K, AKT mTOR, phosphoinositides

## Abstract

Renal failure is a worldwide disease with a continuously increasing prevalence and involving a rising need for long-term treatment, mainly by haemodialysis. Arteriovenous fistula (AVF) is the favourite type of vascular access for haemodialysis; however, the lasting success of this therapy depends on its maturation, which is directly influenced by many concomitant processes such as vein wall thickening or inflammation. Understanding the molecular mechanisms that drive AVF maturation and failure can highlight new or combinatorial drugs for more personalized therapy. In this review we analysed the relevance of critical enzymes such as PI3K, AKT and mTOR in processes such as wall thickening remodelling, immune system activation and inflammation reduction. We focused on these enzymes due to their involvement in the modulation of numerous cellular activities such as proliferation, differentiation and motility, and their impairment is related to many diseases such as cancer, metabolic syndrome and neurodegenerative disorders. In addition, these enzymes are highly druggable targets, with several inhibitors already being used in patient treatment for cancer and with encouraging results for AVF. Finally, we delineate how these enzymes may be targeted to control specific aspects of AVF in an effort to propose a more specialized therapy with fewer side effects.

## 1. Arteriovenous Fistula

**Figure 1:**
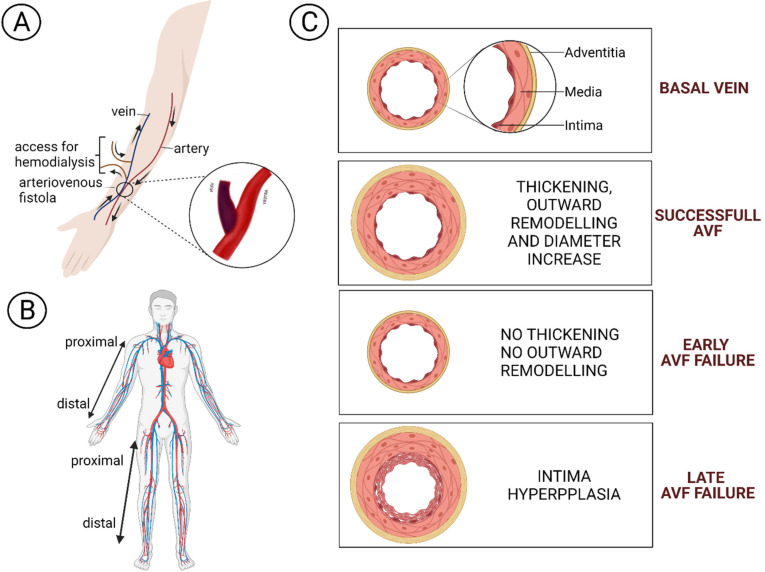
(**A**) An arteriovenous fistula is a connection between an artery and a vein in the arm to facilitate haemodialysis access. (**B**) AVF accesses preferences: first in the non-dominant upper limb, then in the dominant one, and as a final choice in the lower limb. (**C**) Vein wall remodelling in a successful AVF and during early or late failure.

**Figure 2:**
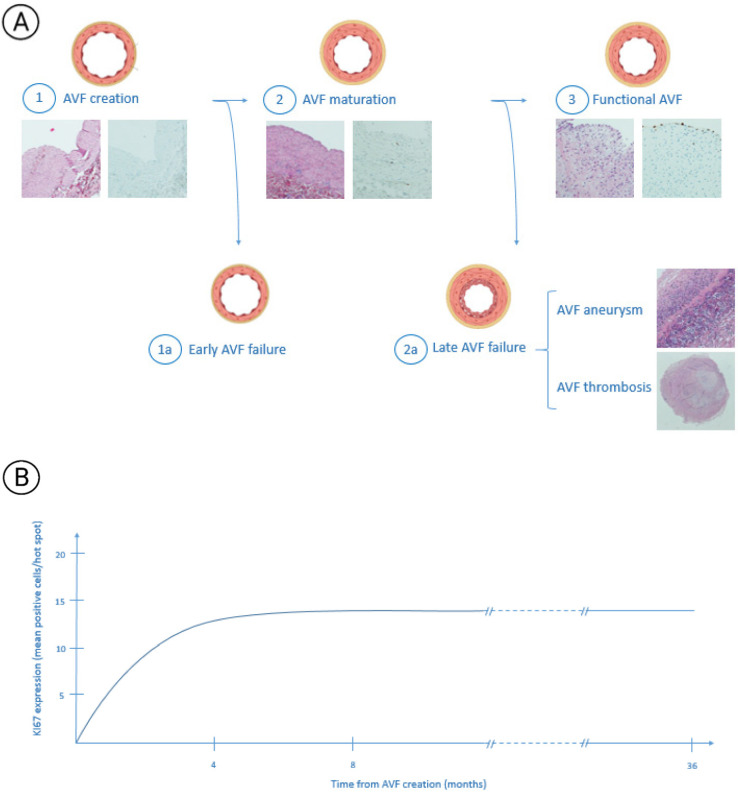
AVF remodelling phases. (**A**) After AVF creation the shear stress and the increase in the venous radial force triggers efferent vein remodelling. In this figure are shown the different phases of this process. The first part of the image shows a normal vein wall (1) that undergoes a maturation process called “arterialization” that leads to myointimal hypertrophy (2). This phenomenon allows for obtaining a mature AVF useful to HD (3). Unfortunately, sometimes this process does not begin or its entity is too poor to develop a usable AVF (1a) or, during the time, it leads to excessive hypertrophy which can result in AVF aneurysm or thrombosis with subsequent AVF failure. Figure 2a shows that histologic stains are obtained by haematoxylin–eosin and immunohistochemistry preparation. All the images were realized by a 10x magnification [12]. (**B**) The figure shows KI67 expression months after AVF creation. It presents a trend of expression during the first 6–8 months from its creation until an expression plateau is achieved that persists during 36 months from AVF creation without important variations [12].

**Figure 3:**
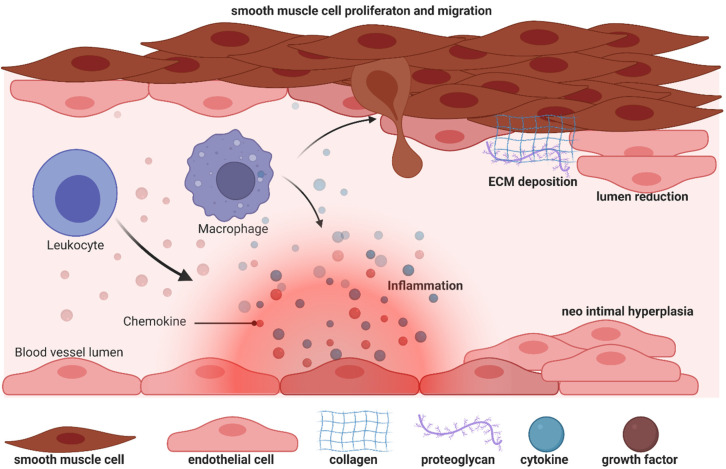
Biology of efferent vein wall of AVF remodelling.

Renal failure is a real increasing public health issue. Over 75 million people worldwide suffer from chronic kidney disease (CKD), more than patients with diabetes, osteoarthritis or depression. Presently, this condition is considered the 12th leading cause of mortality worldwide, and it radically affects the quality of life of a patient. In fact, in 2013 it was listed as one of the top 10 causes of reduced life expectancy or disability-adjusted life years [1,2,3,4]. Haemodialysis (HD) represents the primary modality of renal replacement therapy in people affected by end-stage kidney failure [5,6]. HD requires adequate vascular access, for instance through an arteriovenous fistula, a graft or a tunnelled catheter: these manipulations have a strong impact on patient quality of life, life expectancy, morbidity and mortality [1,2,3,7]. Each form of vascular access for HD presents advantages and disadvantages. Arteriovenous fistula is a connection between an artery and a vein in the arm, starting as distally as possible (Figure 1A) to offer the patient the greatest number of future possibilities for the creation of autologous vascular access. Two types of permanent accesses are used in haemodialysis: (1) a native arteriovenous fistula and (2) a prosthetic fistula created by using synthetic graft or bovine vessels. Native arteriovenous fistulas are considered the gold standard for vascular accesses in HD, with a longer durability, a lower infection rate and a simpler HD management compared to the other vascular accesses [1,2,3,8]. The first option, a more distal AVF, is usually a radial-cephalic fistula created at the wrist of the non-dominant arm (distal AVF) (Figure 1B). The second option is an omero-median, omero-basilic or omero-cephalic fistula created below the elbow; the third option is an omero-basilic superficialized above the elbow. After surgical creation, AVF requires maturation time before use. After AVF creation, the AVF efferent vein begins a new function as its wall becomes more similar to an artery wall and this triggers a new continuous remodelling process [9]. To be successful for haemodialysis, the AVF efferent vein needs to be enlarged and thickened in order for it to sustain both the stronger force of arterial flow compared to venous flow and the intensity of dialysis treatment. Consequently, during its life, the AVF undergoes significant morphological and hemodynamic changes, which are still not completely understood. What is established is that AVF surgical creation triggers wall shear stress and an increase in the venous radial force, by increasing both blood flow and pressure in the AVF efferent vein [10]. Shear stress is defined as the tangential force of the flowing blood on the surface of the blood vessel. It is expressed mathematically by the Poiseuille formula: 4hQ/pr^3^, where h = blood viscosity, Q = blood flow and r = vessel radius [11]. This formula shows that shear stress is directly proportional to blood flow. After AVF creation, the vessel wall adapts itself to the new hemodynamic condition developing hypertrophy, a process known as “arterialization”, where smooth muscle cells (SMC) are the most important cells involved [12]. AVF remodelling is essential for AVF maturation, but it can also lead to complications such as excessive myointimal thickening, and aneurysmatic fistula degeneration with consequent early dysfunction or failure of the AVF (Figure 1C and Figure 2A) [13]. AVF remodelling might have a temporal scan beginning without clear morphological alterations: instead, a significant neointimal hyperplasia usually starts 3.5 months after AVF fistula placement [14] and this AVF remodelling process continues beyond 6 weeks post-operatively, reaching a plateau by three further months [9]. An AVF is considered mature when it includes a flow of at least 600 mL/min, is greater than 6 mm in diameter, is less than 6 mm below the skin and has least 6 cm of the vein for cannulation [15]. Efferent vein wall remodelling can be abnormal and can lead to AVF vein myointimal thickening and/or aneurysmatic fistula degeneration and finally to AVF failure (Figure 2): this process is usually associated with KI67 expression, a known marker of cell proliferation [12]. A particular expression pattern of KI67 in the AVF vein wall has been observed: KI67 expression increases rapidly after AVF creation until it reaches a protein expression plateau. Moreover, matrix metalloproteinase 9 (MMP-9) expression also increases after AVF creation due to shear stress, the uremic environment, inflammation and hypoxia [16], and this increase contributes to an altered matrix deposition together with its degradation, leading to anomalous vascular wall remodelling and neointima formation [16]. Despite notable progress in the knowledge about AVF, causes of its failure are not clear, and the biology of the remodelling process is still not completely understood. Therefore, clinicians and scientists are racing to improve HD techniques and patient outcomes [1,2]. Inflammation also plays a key role in AVF maturation. T cells, macrophages and other immune system cells are involved in the vein wall remodelling with different roles [9]. CD4^+^ T cells guarantee an adequate blood flow and guide outward remodelling, whilst M2 macrophages are essential for developing appropriate vein wall thickening [9]. Macrophages carry out their role by expressing several proinflammatory cytokines and chemokines. They stimulate smooth muscle cell proliferation and migration, resulting in neointimal hyperplasia (Figure 3) [17]. To better understand this complicated process, research can now take advantage of 3D cultures by organoids: for example, Wimmer et al. [18] showed the formation of self-organizing 3D human blood vessel organoids from human pluripotent stem cells (hPSCs) that displayed morphological, functional and molecular features of human microvasculature. Blood vessels formed by organoids can be further cultured in a scalable suspension medium. Notably, in-vitro-differentiated human blood vessel organoids transplanted into immunocompromised mice enter into mouse circulation and participate in the structure of functional arteries, arterioles and veins [18]. Interestingly, organoids have also been used to define processes that underlie the morphological changes observed in histological tissue observations through vascular spheres obtained by human vascular mesenchymal stem cells, 3D symmetric cellular aggregates floating in the culture medium [19].

Even if present-day treatments are improving patient conditions, AVF has a high rate of early or late failure; new or additional treatments are therefore necessary. In this review we focused on the potential roles that phosphoinositides (PPIns) can have in modulating important aspects of AVF, such as cell proliferation, vein wall thickening or inflammation, that could influence new or additional therapies valuable for a more personalized AVF-positive patient life.

## 2. Phosphoinositides

**Figure 4:**
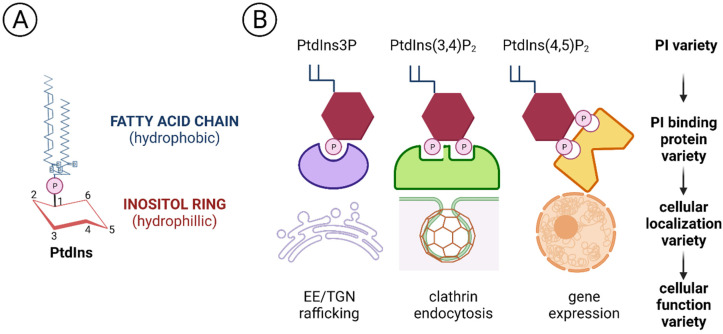
Structure of phosphatidylinositol (PPIns) (**A**) PtdIns is formed by an hydrophilic inositol ring bound to two hydrophobic fatty acid chains. (**B**) Different phosphoinositols can be bound by different specific effector proteins to regulate different cellular functions (modified by [28]).

Phosphoinositides are a group of lipid molecules identified first in the 1950s [20], and which have since been found crucial for many cellular functions, such as proliferation, inflammation, vesicular trafficking and gene expression, so that nowadays it is challenging to imagine a cellular task without PPIns involvement. Phosphoinositides contain a phosphorylated inositol hydrophilic head group linked by a phosphodiester bond to two long hydrophobic fatty acyl tails. The presence of both hydrophilic and hydrophobic groups gives to PPIns the ability to be found in different areas of the cells, such as the hydrophobic cellular/organelle membranes or the hydrophilic cytosol/nucleosol. Furthermore, the inositol head group can be reversibly phosphorylated at the 3-, 4- or 5-OH positions of the inositol ring to generate seven different PPIns: phosphatidylinositol 3-phosphate (PtdIns3P), phosphatidylinositol 4-phosphate (PtdIns4P), phosphatidylinositol 5-phosphate (PtdIns5P), PtdIns3,4-bisphosphate [PtdIns(3,4)P_2_], PtdIns3,5-bisphosphate [PtdIns(3,5)P_2_], PtdIns4,5-bisphosphate [PtdIns(4,5)P_2_] and PtdIns 3,4,5-trisphosphate [PtdIns(3,4,5)P_3_] (Figure 4). This chemical multiplicity is preserved by kinases, phosphatases and phospholipases differently located in the different subcellular compartments, and it is reflected in the distinctive organelles PPIns profiles [21]. For example, PtdIns3P is mainly enriched in early endosomes and is phosphorylated to PtdIns(3,5)P_2_ during endosomal maturation into late endosomes [22], while PtdIns(3,4)P_2_ is found at the plasma membrane, where it can assist clathrin-mediated endocytosis or glucose uptake and insulin signalling [23]. Moreover, PtdIns(4,5)P_2_ is mainly concentrated at the plasma membrane, where it can regulate ion channel activity and vesicle exocytosis, but it can also be localized in the nucleus, where it can regulate chromatin conformation [24], splicing [25] or the epigenetic landscape [26].

PPIns profile diversity is relevant also because different PPIns can recruit compartment-specific effector proteins, which in turn translate the PPIns signal into a functional answer [27,28]. This ability to interact with and thereby regulate many proteins explains why PPIns are significant for so many cellular process [29]. Alterations of PPIns enzymes are associated with many diseases, such as cancer [27,30], neurodegenerative disorders [31], diabetes [32] or myotubular myopathies [33]. In this review we focus on PI3K/AKT/mTOR signalling as the key phosphoinositide players to illustrate their potential value for AV fistulas.

## 3. PI3K/AKT/mTOR Axis

Phosphoinositide 3-kinases are enzymes which catalyse the phosphorylation of one or more inositol phospholipids on the 3-position of the inositol ring [34]: in mammalian cells there are eight PI3K isoforms that can be divided into three classes based on common substrate specificity, structural similarities and an analogous mechanism of activation.

Class I PI3Ks are well-characterized enzymes that respond to signals received through cell surface receptors such as activated tyrosine kinase receptors (RTKs), G protein-coupled receptors (GPCRs) and monomeric small GTPases [35,36]. Class I PI3Ks are heterodimers made by a regulatory subunit and a catalytic subunit [37]. Once stimulated, the receptor becomes activated and its intracellular portion is able to be bound by a regulatory subunit, which in turn activates the catalytic subunits that phosphorylate PtdIns(4,5)P_2_ into PtdIns(3,4,5)P_3_ at the lipid membranes [38]. Among the different isoforms, PI3Kα and PI3Kβ are relatively ubiquitously expressed, while the PI3Kγ and PI3Kδ isoforms are enriched mainly in hematopoietic cells, such as leukocytes [36,39].

Class II PI3Ks have distinguishing, non-overlapping functions and are involved mainly in endocytosis, membrane dynamics and trafficking [40]. Class II PI3Ks are monomers present in three isoforms (PI3K-C2α,-C2β and -C2γ): in contrast to Class I PI3Ks, they are not stimulated by a cell surface receptor, but are activated by internal cell signalling [41]. PI3KC2α and PI3KC2β are expressed ubiquitously, while PI3KC2γ is present mainly in the liver [42].

Class III PI3K is represented by a single isoform known as vacuolar protein sorting 34 (Vps34) occupied in controlling vesicular endosome-lysosome sorting, which might induce pathogen killing, antigen processing and immune cell survival [35,43].

The three classes generate diverse lipid products, and therefore recruit different downstream effectors. Notably for this review, Class I, but not Class II, phosphorylates PtdIns(4,5)P_2_ into PtdIns(3,4,5)P_3_, which in turn drives the localisation of protein kinase B (PKB, also known as AKT) to the plasma membrane together with its upstream regulatory kinases. Co-localisation at the plasma membrane essentially increases the effective concentration of AKT and its kinases, thereby increasing the likelihood of interaction. In addition, interaction of 3-phosphoinositide-binding pleckstrin homology (PH) domain of AKT with PtdIns(3,4,5)P_3_ also induces a conformational switch which enables AKT phosphorylation by its upstream activators. AKT is a serine/threonine kinase with a multitude of substrates and targets; this in part explains the relatively wide spectrum of effects of PtdIns(3,4,5)P_3_ signalling on downstream pathways.

Another PI3K effector can be mTOR. mTOR exists in two multi-protein complexes, mTORC1 and mTORC2, with distinct subunit composition, substrate selectivity and activity and sensitivity to sirolimus (rapamycin). A well-established substrate of mTORC2 is AKT, phosphorylated in its Ser473, in addition to protein kinase C (PKC) isoforms and serum- and glucocorticoid-regulated kinases (SGKs) [34]. mTORC1 phosphorylates several substrates that induce anabolic metabolism to sustain growth and proliferation [34]. mTORC1 action can be induced by proliferating stimuli via PI3K/AKT, RAS/ERK and other signalling cascades [44].

Overall, the PI3K/AKT/mTOR axis transmits important signals that regulate a variety of physiological processes in virtually all tissue types studied to date (which have been extensively described in literature) such as proliferation, apoptosis, inflammation cellular migration and immune response, and its dysregulation is associated with development of various diseases including cancer, diabetes and autoimmunity: Here we describe highlights of the PI3K/Akt7mTORC1 axis in arteriovenous fistula patency.

## 4. PI3K/Akt/mTOR Pathway in AVF

Unfortunately, AV fistulas fail to mature in 30–50% of cases (early failure) and a mature correct AV fistula fails in one year (late failure) in 40% of cases, reflecting a dramatic clinical outcome and the urgent need for more a accurate understanding of the mechanisms that impair the fistula. During AVF vein remodelling, a complex scenario takes place involving interconnected processes such as inflammation, VSMC proliferation and consequently neointima hyperplasia, extracellular matrix deposition and adventitia remodelling: in all these functions PI3K/AKT/mTOR signalling pathways can be implicated with different and even contrasting outcomes.

VSMCs dedifferentiation can be induced by growth factors and inflammatory cytokines locally released after vascular injury [16]: among these, platelet-derived growth factor (PDGF) is one of the most powerful mitogens that can induce a “phenotypic switch” from a contractile to a proliferative state, an essential step for neointima hyperplasia. PDGF-induced VSMC proliferation [45,46] is mediated by a signalling cascade that includes PI3K/AKT, mTOR/p70S6kinase and MEK1/ERK signalling downstream of the insulin receptor stimulus. Moreover, by interfering with dysregulated insulin receptor signalling, Zhao et al., postulated new therapeutic options to prevent VSMC proliferation both in nondiabetic and in diabetic states [45]. Therapeutic possibilities have also been studied by Chen et al., who analysed the effect of dasatinib, an orally bioavailable protein tyrosine kinase inhibitor, currently undergoing clinical trials in cancer patients [47]. Dasatinib is a potent PDGFR inhibitor at low nanomolar concentrations: comparison between dasatinib and imatinib showed that the former was able to decrease VSMC proliferation and inhibit PI3K/AKT at lower concentrations in A10 rat aortic smooth muscle cell lines [47]. To contrast VSMCs proliferation another interesting compound can be n-butylidenephthalide (BP), which was able to reduce PDGF-induced phenotypic switches both in vitro and in an AVF rat model, and it may contribute to vascular protection [48]. Yang et al., in fact, showed that BP can exert an anti-migrating effect, thus limiting the movement of VSMC from the media to the intima, and can also promote cells’ quiescent state by arresting cells in the G_0_/G_1_ cell cycle phase [48]. In rat fistula, BP can increase lumen size and decrease thrombus formation. Notably, these important effects are realized by the induction of AMPK and concomitant reduction of mTOR phosphorylation and signalling.

The PI3K/AKT/mTOR axis is also involved in extracellular matrix (ECM) formation: in fact, inflammation-released cytokines and growth factors can also influence extracellular matrix (ECM) degradation, which in turn helps VSMC infiltration and new matrix deposition. Notably, matrix metalloproteinase 9 (MMP-9) expression is upregulated in vascular inflammation and participates in vascular remodelling [16,49]. Recently, Shih et al. used MMP-9 knock-out mice with CDK and observed a decrease in inflammation, an increase in AVF lumen diameter, and attenuated neointima formation due to a concomitant reduction in collagen factor and smooth muscle cell proliferation, compared to wt CDK mice [16]. Since AKT and ERK phosphorylation increases in neointimal lesions in AVF, the authors showed that, by western blot experiments, the AKT and ERK phosphorylation increase is attenuated by MMP-9 knockout [16]. Importantly, PI3K/AKT/mTOR signalling repression can be related to a decrease in the inflammation, migration and proliferation of VSMC cells.

Since this AKT/mTOR involvement in cell growth and inflammation, inhibition of this signalling cascade by rapamycin treatment has been tested in diabetic patients and has shown efficacy in reducing neointimal hyperplasia by decreasing migration of smooth muscle cells and rapamycin. It is now currently used for patient therapy [50]. Dardik and colleagues extended involvement of AKT/mTOR signalling showing that it is upregulated in venous remodelling during both graft adaptation and AVF maturation [51]. Notably, amounts of Eph-B4 receptor as well as its ligand Ephrin-B2 increases in the vein wall during AVF maturation, and its signal is mediated through AKT activation [52]. Additional studies showed that rapamycin treatment caused a decrease in wall thickening due to diminished extracellular matrix deposition combined with a reduction in the proliferation of endothelial cells, muscle cells and macrophages [53]. Molecular investigation of this process demonstrated that rapamycin reduced activation of AKT/mTORC1 but not of mTORC2, and this was reflected in a reduction in the amounts of downstream targets of mTORC1, P70S6k and 4EPB1 [53], reinforcing the concept that inhibition of the AKT/mTORC1 axis with rapamycin reduces the pathologic venous remodelling that is associated with AVF failure.

**Figure 5:**
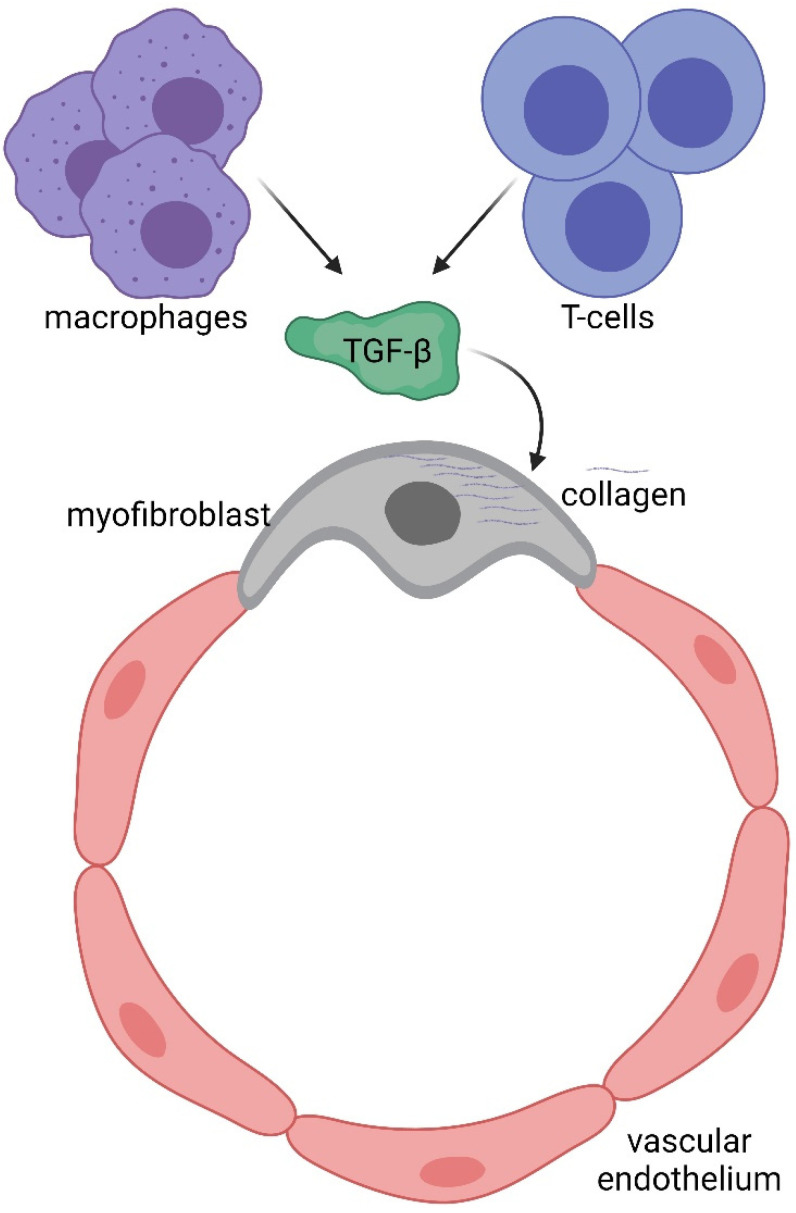
Immune system involvement in endothelial-to-mesenchymal transition (EndoMT).

To understand potential pathogenic steps in AVF failure, many studies have highlighted that remodelling of adventitia is caused also by the infiltration of myofibroblasts [54] accumulated in the adventitia. These myofibroblasts are responsible for fibrogenesis since they become a major source of collagen, producing an excess of the extracellular matrix at the base of the initiation and progression towards a pathological fibrotic vessel [55]. The exact source of the mesenchymal cells that produce collagen or other extracellular matrix components and become a central bulk of the mesenchymal cells participating in the fibrotic process is still not clear. Activated fibroblasts can originate from resident or vascular adventitial fibroblasts that pass from being quiescent to proliferative in response to signals received through the inflammation process [56]. They can also originate from epithelial cells that become myoblasts in response to signals such as transforming growth factor β (TGF-β) [57], and drive epithelial-to-mesenchymal transition (EMT) or endothelial-to-mesenchymal transition (EndoMT). In fact, EndoMt has emerged as a transdifferentiation biological process that induces endothelial differentiated cells to acquire a different (mesenchymal or myofibroblastic) phenotype and so able to produces mesenchymal proteins such as SMA, vimentin, and Type I collagen (Figure 3 and Figure 5). This transdifferentiation can be activated by the PDGF receptor along with a marked increase in the phosphorylation of Akt and ERK, two key kinases in PDGFRβ signalling [46]. Inhibition of this signalling cascade can reduce the presence of myofibroblasts that were the main cell type associated with the activation of p-PDGFRβ. The EndoMt process can be stimulated also by TGF-β released by lymphocytes and macrophages present due to the inflammatory state [55]. Notably, macrophages and lymphocytes play key and controversial roles in AVF inflammation and thus in AVF failure. High levels of inflammation (measured by c-reactive protein and fibrinogen) [58] are associated with AVF failure; however, depletion of macrophages and T cells also results in AVF failure. This controversial relationship can in part be explained by the different types and roles of immune cells in vascular wall formation and thus AVF patency and maturation (clearly reviewed in [9]).

The immune system consists of a sophisticated network of cells which recognise and eliminate foreign threats to our body and provide immunological memory against external pathogens. Among the different immunological cell types, T cells provide adaptive immunity (acquired immune system), while macrophages (circulating monocytes that become resident next to the vessel wall) work as innate immunity. The two arms collaborate together during defensive processes such as inflammation. During AVF remodelling in the early phase, T cells contribute positively to augmented blood flow and outward remodelling, but unfortunately T regulatory (Treg), T helper2 (Th2) and M2 macrophages are related to late AVF failure [9]. Similarly (but also on the contrary), during the late phase, T helper1 (Th1) and M1 macrophages inhibit neointimal hyperplasia and thus enhance AVF patency, but unfortunately their presence is associated with primary AVF. Inhibition of AKT/mTORC1 signalling by sirolimus is now being used in clinical patients to help AF patency, probably by regulating Treg cells [59]. It is possible that in the future different inhibitors will be used to selectively limit specific cells of the immune system and improve clinical outcomes.

## 5. Conclusions

Although much progress has been made in understanding the role of PI3K/AKT/mTOR signalling in AVF, many questions are still not answered. PI3K/AKT/mTOR signalling performs a multifaceted and often contrasting function in the regulation of AVF, and the effect of inhibiting PI3K/AKT/mTOR is dependent on the context of activation, i.e., regulating different cells of the immune system. As for cancer research, the features regulating the opposing roles of PI3K/AKT/mTOR signalling have still not been plainly comprehended, and they deserve further studies. Many data are now suggesting that these enzymes are critical for intima and adventitia neoplasia, EndoMt transition and reduction in inflammation; the intricacy of PI3K/AKT/mTOR signalling organization can suite the challenge for discovering new therapeutic uses of PI3K/AKT/mTOR inhibitors in AVF. Animal models and early clinical trials show great potential in the therapeutic targeting of this pathway in AVF maturation, but may not always lead to clinical successes.
